# A Real-Time Thermal Sensor System for Quantifying the Inhibitory Effect of Antimicrobial Peptides on Bacterial Adhesion and Biofilm Formation

**DOI:** 10.3390/s21082771

**Published:** 2021-04-14

**Authors:** Tobias Wieland, Julia Assmann, Astrid Bethe, Christian Fidelak, Helena Gmoser, Traute Janßen, Krishan Kotthaus, Antina Lübke-Becker, Lothar H. Wieler, Gerald A. Urban

**Affiliations:** 1 Department of Microsystems Engineering (IMTEK)—Laboratory of Sensors, University of Freiburg, 79110 Freiburg, Germany; helena.gmoser@venus.uni-freiburg.de (H.G.); krishan.kotthaus@saturn.uni-freiburg.de (K.K.); urban@imtek.de (G.A.U.); 2 Institute of Microbiology and Epizootics, Freie Universität Berlin, 14163 Berlin, Germany; j.assmann@fu-berlin.de (J.A.); Astrid.Bethe@fu-berlin.de (A.B.); antina.luebke-becker@fu-berlin.de (A.L.-B.); WielerLH@rki.de (L.H.W.); 3 Robert Koch Institute, ZBS4 Advanced Light and Electron Microscopy, 13353 Berlin, Germany; 4 Bovicare GmbH, 14473 Potsdam, Germany; fidelak@bovicare.de; 5 RIPAC-Labor GmbH, 14476 Potsdam-Golm, Germany; tjanssen@ripac-labor.de; 6 Robert Koch Institute, 13353 Berlin, Germany

**Keywords:** thermal biosensor, AMPs, measurement in real time, white light interferometry

## Abstract

The increasing rate of antimicrobial resistance (AMR) in pathogenic bacteria is a global threat to human and veterinary medicine. Beyond antibiotics, antimicrobial peptides (AMPs) might be an alternative to inhibit the growth of bacteria, including AMR pathogens, on different surfaces. Biofilm formation, which starts out as bacterial adhesion, poses additional challenges for antibiotics targeting bacterial cells. The objective of this study was to establish a real-time method for the monitoring of the inhibition of (a) bacterial adhesion to a defined substrate and (b) biofilm formation by AMPs using an innovative thermal sensor. We provide evidence that the thermal sensor enables continuous monitoring of the effect of two potent AMPs, protamine and OH-CATH-30, on surface colonization of bovine mastitis-associated *Escherichia* (*E.*) *coli* and *Staphylococcus* (*S.*) *aureus*. The bacteria were grown under static conditions on the surface of the sensor membrane, on which temperature oscillations generated by a heater structure were detected by an amorphous germanium thermistor. Bacterial adhesion, which was confirmed by white light interferometry, caused a detectable amplitude change and phase shift. To our knowledge, the thermal measurement system has never been used to assess the effect of AMPs on bacterial adhesion in real time before. The system could be used to screen and evaluate bacterial adhesion inhibition of both known and novel AMPs.

## 1. Introduction

The discovery of the antimicrobial effect of the fungus *Penicillium* in 1928 by Alexander Fleming was a milestone for human and veterinary medicine, allowing the effective treatment of bacterial infections [1]. Different antibiotic substance classes with bactericidal or bacteriostatic effect are available today. However, the extensive administration of antibiotics in human and veterinary medicine increased the selective pressure on bacteria [2]. Consequently, antimicrobial resistance (AMR) of pathogenic bacteria increased over the decades, with potentially fatal consequences in the case of infections in human and animal patients [3,4]. Rising awareness with respect to AMR is needed in the medical sector but also in the general public to promote the prudent use of antibiotics and not least to spur development of suitable alternatives to common classes of antibiotics [5].

Various bacteria organize themselves in biofilms, a microbial community surrounded by a viscous matrix of extracellular polymeric substances (EPSs), including constituents such as polysaccharides, proteins, extracellular DNA, and lipids [6,7]. The cells within a biofilm adhere to one another and the growing bacterial community can reside on biotic or abiotic surfaces [8]. In contrast to planktonic bacteria, living in a biofilm provides several key advantages to the bacterial colony as a whole, including protection against environmental effects, like antibiotics or disinfectants, and the accelerated transport of nutrients and signal molecules inside the biofilm [6,9,10]. Within the biofilm, a cell can become 10–1000 times more resistant to the effects of antimicrobial agents compared to its planktonic state (reviewed in [9]). The lifecycle of a biofilm starts with the adhesion of planktonic bacteria to a surface. The bacteria form aggregates, start to produce EPSs, and form a mature biofilm, which releases planktonic bacteria actively or passively (reviewed in [10]).

Studies investigating the formation and development of bacterial communities are of medical importance since 60% of persistent infections are caused by biofilms [11]. Bacterial growth on and in different types of indwelling catheters and implants is typical, but periodontitis and endocarditis are also often caused by bacteria residing in biofilms (reviewed in [12,13]). Consequently, biofilm-associated infections are a clinical problem, not only in human but also in veterinary medicine. Bovine mastitis, for instance, is the most important biofilm-associated disease in the dairy industry, which causes major economic losses while posing a threat to animal health and welfare [14,15].

Biofilms are of particular importance concerning AMR in pathogenic bacteria. Genetic information is shared by horizontal gene transfer between bacteria co-existing in a biofilm [16], allowing them to exchange and implement information on AMR and tolerance towards other (environmental) influences. Even a low concentration of antimicrobial substances can increase these self-defense mechanisms [17] and thus bacterial resistance [18].

Antimicrobial peptides (AMPs) consist of cationic and hydrophobic sequences of 12–100 amino acids in length, structured as α-helix, β-sheet, mixed, or linear conformations [16,17]. Currently, more than 5000 AMPs are known, including synthetic agents as well as those produced by animals, bacteria, fungi, and plants [17]. There are many possibilities to utilize the effects of AMPs towards unwanted bacteria, including decontamination of fluids and, when associated with a surface, reduction of bacterial adherence [19]. AMPs are effective against a variety of bacteria, including multidrug-resistant (MDR) variants, but also fungi and viruses (reviewed in [18,20]) [21]. Even at very low concentrations, some AMPs can cause the destruction of microorganisms [22]. The complex mechanisms of the AMP effect on bacteria are currently the subject of intensified research. The charged amino acids of the AMPs interact with the hydrophilic head groups of the membrane-associated phospholipids. In addition, the interaction of hydrophobic domains of the peptides, the phospholipids, and of the bacterial membrane lead to a transfer of the AMPs into the membrane and lead to the lysis of the membrane [23]. Furthermore, AMPs can perforate the cytoplasmic membrane [24] or inhibit certain intracellular molecules [25]. This results in lysis of the cell or cell death [18]. Thus far, AMPs seem to lack typical receptor interactions, which are characteristic for different antibiotic classes. Thus, AMPs belong to a novel class of anti-infective agents, which could potentially substitute antibiotics and solve concomitant problems caused by multidrug resistance [26]. Currently, there are only a few approved AMPs for clinical applications (reviewed in [27]).

Various laboratory methods for measuring the lifecycle and structure of biofilms have been established, including the staining of bacteria sticking to a microwell plate [28] and characterization of the polymeric conglomerates by optical and electron microscopic methods [29]. However, these methods do not allow the continuous monitoring of bacterial adhesion in real time. Considering these most frequently used methods to characterize bacterial adhesion, utilizing a thermal sensor offers significant advantages such as monitoring and measuring of the dynamic processes of the formation, inhibition, and removal of bacterial adhesion.

An overview of other biofilm monitoring methods with their advantages and disadvantages is given in Table 1.

The process of bacterial adhesion can be monitored by a sensor detecting the propagation of surface acoustic waves (SAWs) generated by an interdigital transducer (IDT) on a zinc oxide (ZnO) piezoelectric layer [30,31,32]. Another technique employs the principle of electrochemical impedance spectroscopy (EIS), which measures impedance changes induced by bacterial adhesion by using an interdigital electrode structure [33,34]. Measurement of adherent mass variation by the quartz crystal microbalance (QCM) method is another approach to monitor bacterial adhesion by utilizing the change in resonance frequency of a piezoelectric quartz oscillator [35].

Isothermal microcalorimetry (IMC) measures the heat produced by bacterial metabolism in the adhering bacteria [36,38]. A further indirect measurement of adherent bacteria accumulation depends on the change in thermal resistance using a heating resistor and a temperature measurement diode in contact with the growth medium with an external feedback circuit to keep the temperature constant while recording the power consumption of the resistors [39]. Optical sensors for bacterial adhesion are based on different principles: Intrinsic fluorescence of the amino acid tryptophan, for instance, can be excited by a UV LED light [40]. Another measuring principle is based on the diffraction pattern of adherent cells illuminated by an RGB LED recorded with a complementary metal oxide semiconductor (CMOS) sensor [41]. Refractive index tapered fiber optic biosensors that detect changes in the evanescent waves induced by bacterial adherence have also been successfully used [35,43].

A new sensor based on heat transfer by alternating current (AC) thermal excitation is not susceptible to baseline drift caused by electrochemical processes because of its completely passivated fabrication, in contrast to most EIS designs. In this case, the thermal sensors are optimally suitable for measurements in liquids of high ionic strength in real time. None of these measurement systems allows compact long-term bacterial adherence determination in real time without knowing the exact thermal properties, except for the AC method [44].

The objective of this study was the real-time measurement of bacterial adherence and the effect of AMPs as adherence-inhibiting substances using a thermal sensor. Thermal waves are generated by a chromium heater, excited with a sinusoidal signal (40 Hz). The resulting temperature changes are detected by an amorphous germanium thermistor [45,46,47]. To determine the thermal properties of fluids, the amplitude variation and phase shift of the measured temperature oscillations over time are analyzed. The phase shift is used to determine the thermal conductivity λ, which is in a linear relation to the product of specific heat capacity c_p_ and density ρ of the analyzed fluid [47,48,49].

The sensors are constructed using thin-film technology deposited on a thermal insulating membrane. In contrast to EIS, the full passivation of the sensors provides high resilience to potential measurement errors, for instance, induced by changes in the ionic composition of the medium as a result of metabolic bacterial activity [48]. In addition, the thermal measuring method is also completely insensitive to the turbidity of the medium due to growing planktonic cells, as opposed to optical detection. The sensors are also reusable after a cleaning process compared to the limited reusability of the SAW sensor [44,50].

## 2. Materials and Methods

### 2.1. Bacterial Culture Strains and Antibiotic Susceptibility Testing

The two reference strains *Escherichia* (*E.*) *coli* (ATCC25922) and *Staphylococcus* (*S.*) *aureus* (ATCC29213) were obtained from the American Type Culture Collection (ATCC).

Bacterial strains *E. coli* (IMT37453) and methicillin-resistant *S. aureus* (MRSA) (IMT37556) isolated from cases of bovine mastitis (Bavaria, Germany, 2014 and Saxony-Anhalt, Germany, 2015) were additionally used in this study. Antibiotic susceptibility testing was performed using the MICRONAUT-S Mastitis 3 system (Merlin GmbH, Rüsselsheim, Germany) according to the manufacturer’s instructions. The tested antimicrobial agents and their concentrations are listed in Table 2. They were evaluated according to Clinical and Laboratory Standards Institute (CLSI) guidelines and Feßler et al. [51,52].

### 2.2. Antimicrobial Peptides (AMPs)

Two different AMPs were purchased. The AMP protamine, originally identified in salmon sperm (*Salmo salmine*) [53], was partly expressed, isolated, purified, verified, and utilized for the measurements (ILBC GmbH, Potsdam, Germany) (Table 3). It was produced using recombinant DNA technology, and isolated and purified using reverse column chromatography through high-performance liquid chromatography (HPLC) as described in patents [54]. The AMP OH-CATH-30, a fragment of OH-CATH missing 4 N-terminal amino acids, originally identified in king cobra (*Ophiophagus hannah*) [55], was chemically synthesized and purchased (genecust, Ellange, Luxembourg). The peptides were dissolved in deionized (DI) water and stored at −20 °C, followed by further individual dilution if necessary.

### 2.3. Antimicrobial Assay—Determination of Minimal Inhibitory Concentrations (MICs) of Different AMPs

Minimal inhibitory concentrations (MICs) of the used peptides were determined against Gram-negative *E. coli* (IMT37453, ATCC25922) and Gram-positive *S. aureus* (IMT37556, ATCC29213) by a standard microdilution method using microwell cell culture plates as previously described [56].

Briefly, different concentrations of each AMP (2000 µg/mL to 15.63 µg/mL in two-fold serial dilution steps) were prepared in Mueller–Hinton-1 (MH1) medium and 50 µL were distributed into a 96-well microtiter plate (Thermo Scientific Heraeus, Schwerte, Germany). Single colonies were inoculated in MH1 medium, diluted to approximately 2 × 10^5^ colony-forming units (CFU)/mL, and 50 µL of the prepared inoculum were added to each well, achieving a final testing volume of 100 µL and a final testing concentration range of OH-CATH30 and protamine between 7.8 µg/mL and 1000 µg/mL. Bacteria cultured in MH1 medium without antimicrobial substances were used as a positive control and sterile MH1 medium was used as a negative control. After 24 h of incubation at 37 °C, the absorbance at OD_600nm_ was measured after shaking to ensure homogenous distribution (Synergy HT Microplate Reader, BioTek, Bad Friedrichshall, Germany). The first concentration with no visible bacterial growth was determined as the MIC for this AMP in combination with the tested strain.

### 2.4. Whole Genome Sequencing

The two mastitis isolates were whole-genome sequenced (WGS) using Illumina MiSeq 300 bp paired-end sequencing with an obtained coverage >90×. Raw reads were used for de novo assembly into contiguous sequences (contigs) and subsequently into scaffolds using SPAdes v3.12 [57]. Assembled draft genomes of the isolates were annotated using Prokka [58]. Genomic data were analyzed with ResFinder-2.2 (threshold: 90% ID, 80% minimum length) [59].

### 2.5. Thermal Sensor Fabrication Process

A standard silicon wafer (4 inches, 525 µm) coated on both sides with a thermal silicon dioxide layer (0.4 µm) followed by a low-pressure chemical vapor deposition (LPCVD) silicon nitride layer (0.11 µm) was used as supporting material for the membrane (600 µm × 700 µm). The heating structure (Cr, 0.1 µm), the thermistors (a-Ge, 0.2 µm), and the conducting lines and paths (Ti, 0.02 µm/Au, 0.17 µm/Ti, 0.02 µm) were structured by lithography, physical evaporation, and lift-off processes. On the front side, a plasma-enhanced chemical vapor deposition (PECVD) silicon nitride layer (0.72 µm) for passivation and encapsulation of previous structures was deposited. It can be applied at a temperature of 120 °C, which was necessary to prevent crystallization of the amorphous germanium thermistors.

The backside of the sensor was used for consecutive measurement because the PECVD silicon nitride layer on its top contains pinholes, which might promote oxidation of germanium in a liquid medium. The chrome heating structure, titanium adhesion-promoting layers, and gold contacts would not significantly be affected by oxidation, as chromium and titanium form a stable passivating oxide and gold has high corrosion resistance. After opening the silicon dioxide/nitride layer at the backside by reactive ion etching (RIE), the silicon was wet etched by 30% potassium hydroxide (KOH) to uncover the resulting membrane (1.2 µm). Further information about the sensor geometry and detailed fabrication processes can be found in previous publications [48,60].

The heater was made of chromium because of its low temperature coefficient of resistance (TCR) (0.01%/K) [61] and its total resistance at room temperature is 3–6 kΩ. It has a length of 1.6 mm, a width of 6 µm, and a thickness of 100 nm. Amorphous germanium was chosen for the thermistors because of its high TCR (−2.3%/K), resulting in a resistance at room temperature of 0.5–1.2 MΩ. The dimensions of the thermistor are 574 µm × 6 µm × 200 µm (length × width × thickness) [45,46]. Further, as shown in previous applications of the same sensor, the resolution of temperature measurements was limited to 0.48 mK by noise at a response time of 3 ms by a bandwidth of 4 kHz [46]. Only the central thermistor was used in this application for measuring the temperature oscillations caused by the heater.

### 2.6. Chip Layout

**Figure 1:**
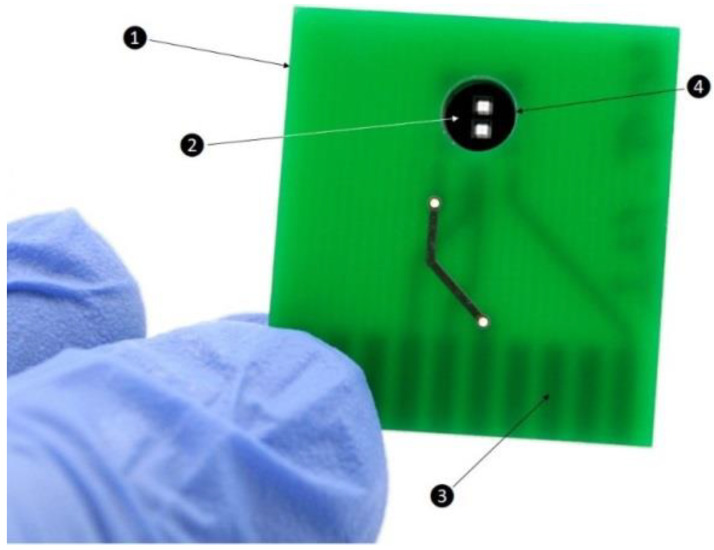
Layout of the chip for the thermal measurement. (1) Frontside of printed circuit board, (2) sensor die bonded on the back side, (3) integrated edge connector (visible on the back), (4) drilled hole.

The total dimensions of the printed circuit board (PCB) (Figure 1) were 30 mm × 27.7 mm × 1.6 mm (width × high × depth). The sensor die was attached at the backside with a one-component epoxide resin (Structalit 8804, Panacol, Germany), cured for 10 min at 120 °C and electrically connected via aluminum bonding wires (25 μm). To connect the sensor and the edge connector with the traces located on the backside (Figure 1) and avoid crossing traces, one of the traces was located across the front side. The printed circuit board had an integrated edge connector, connected to the preamplifier and power supply board. A hole was drilled with an inside diameter size of 5.35 mm to make the membrane accessible for the measurement.

### 2.7. Sample Preparation for Sensor Application

An overnight culture grown on MH1 agar was used to prepare the inoculum. In brief, colony material was suspended in MH1 broth to reach a final concentration of 1 × 10^5^ CFU/mL. One hundred microliters of the inoculum were then immediately placed in the sample chamber of the thermal sensor and the lid was closed to prevent sample evaporation. The measurement setup was placed in an incubator at 37 °C without shaking, connected to the amplifier circuit board and the data recording was started after the thermal equalization period. For AMP experiments, the respective MIC values determined as well as one value above the MIC were used. The MIC values were rounded up to whole numbers to ensure better comparability and easier handling in the thermal sensor experiments. The AMPs were added to the sample chamber at the beginning of the measurement.

### 2.8. Cleaning and Sterilization Protocol of the Thermal Sensor and the Sensor Connection Setup

A cleaning and sterilization protocol was developed to remove adherent bacteria from the sensor membrane and avoid cross-contamination between experiments. Peracetic acid (PAA) (15%) was applied to clean and disinfect the device with a contact time of two hours [19]. After rinsing with deionized (DI) water, the sensors were dried using hot air (100 °C/24 h) and slowly cooled down to room temperature. Thereafter, the resistances of the heating structure and the thermistor were measured again to verify the lack of functional alterations. Furthermore, the thermal sensor connection setup components were autoclaved for 20 min at 121 °C [60].

### 2.9. White Light Interferometry

Selected strains were incubated for 24 h at 37 °C under static conditions in the sample chamber of the thermal sensor connection setup, as described before. After careful removal of the supernatant and disassembling of the sample chamber from the thermal sensor, the chip was dried for one hour at 37 °C to remove residues of the liquid. Subsequently, 3D images of the bacterial structures of the biofilm were taken with the Zygo newView 9000 (AMETEK Germany GmbH, Weiterstadt, Germany). The measurements’ raw data were determined by the graphic program Gwyddion (Department of Nanometrology, Czech Metrology Institute, Czech Republic).

### 2.10. Thermal Sensor Connection Setup

The PCB was placed in a bracket made of polyether ether ketone (PEEK), which consists of three parts (top cover, central part, and bottom cover): A top cover to prevent fluid loss due to evaporation with an O-ring (MVQ 50 red 8 mm × 2 mm, Arcus GmbH, Germany) to improve the sealing against oxygen access, a central part with a cylindrical sample chamber (141 μL) with an indentation for fixation of the PCB and another smaller indentation to accommodate an O-ring to seal between the sensor die and the sensor board, and four threaded holes for mounting screws for the top cover and bottom cover.

**Figure 2:**
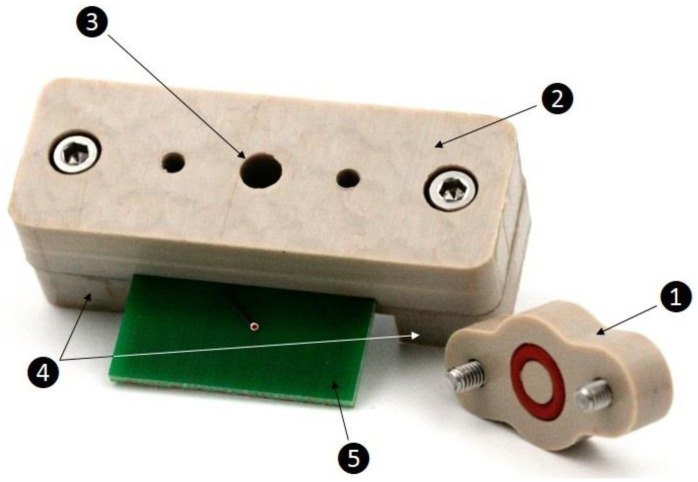
Thermal sensor connection setup. (1) Top cover with an O-ring and two screws, (2) central part with an O-ring on the underside (not shown) and two screws, (3) cylindrical sample chamber with a volume of 141 µL, (4) bottom part divided in two pieces, (5) printed circuit board with bonded sensor die (not shown).

The bottom part was divided into two pieces, which pressed the PCB onto the central part, and was connected via two screws (Figure 2).

The measurement setup is reusable; all parts are made of durable, biocompatible materials and are completely autoclavable.

**Figure 3:**
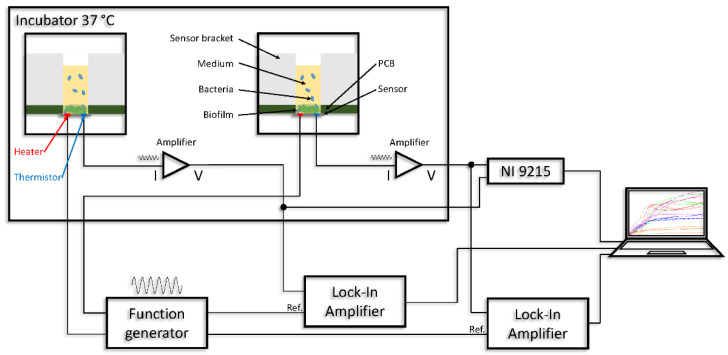
Thermal sensor measurement setup with all components for carrying out the experiments. Integrated function generator shown separately for clarity.

The thermal sensor measurement setup (Figure 3) consists of an incubator (ES-20, Biosan, Riga, Latvia) in which the temperature was set to 37 °C to provide a stable thermal environment. Two sensor brackets, shown in Figure 2, and two preamplifiers with power supply boards were placed in the incubator. The preamplifier and power supply boards in one were used to provide a stable voltage of −250 mV. The signal from the thermistor was converted into a voltage signal by a trans-impedance amplifier (current-to-voltage converter). The excitation voltage, the measurement data, and the power supply voltage were fed in and out through the incubator wall with cable feedthroughs using shielded coaxial cables (RG303, Harbour Industries, Shelburne, VT, USA). Outside the incubator, two lock-in amplifiers (SR-810 und SR-830, Stanford Research Systems, Sunnyvale, CA, USA), a power supply, two analog-to-digital (ADC) converters (NI 9215, National Instruments, Austin, TX, USA), and a laptop were placed. The power supply was connected to the preamplifier and power supply boards to provide a stable voltage. The excitation of the heater was generated by the integrated function generators of the lock-in amplifiers. The unmodified sinusoidal signal applied to the heater was also connected to the reference input of the lock-in amplifier. The lock-in amplifiers were connected to a laptop via a GPIB-USB adapter (488-USB2, ICS Electronics, Hayward, CA, USA). The signals of the preamplifier were routed to the two digital lock-in amplifiers, converted to a DC voltage, and the signal amplitude of the 40 Hz signal was filtered out, digitalized, and sent to the laptop. The digitized sinusoidal signal was read out, sent through a low-pass filter, and written to a file by a program written in LabVIEW (Version 2010, National Instruments, Austin, TX, USA). The program also read out the previously measured resistance of the heating structure of the sensor from a file and adjusted the voltage of the excitation signal so that its power was 0.5 mW. As a result, thermal influence on the adhering bacteria was prevented. More details about the theoretical measurement setup can be found in the publication of Diego F. Reyes-Romero et al. [44].

## 3. Results and Discussion

### 3.1. Results of MIC Experiments and Whole-Genome Sequencing

The *S. aureus* strain IMT37556 showed phenotypic AMR to erythromycin, oxacillin, penicillin G, and pirlimycin. Methicillin resistance was proven by the resistance against oxacillin and the presence of the *mecA* gene. Consequently, IMT37556 was determined to be resistant against all β-lactam antibiotics. Furthermore, IMT37556 harbored the β-lactamase gene *blaZ*-like and the two tetracycline efflux pumps *tet*(*K*) and *tet*(*M*).

The *E. coli* strain IMT37453 showed AMR to ampicillin, cefazolin, cefoperazone, cefquinome, and marbofloxacin. The WGS revealed the β-lactamase genes *bla*_OXA-1_ and *bla*_TEM-1A_, the chloramphenicol acetyltransferase *catA*1, the trimethoprim resistance gene *dfrA*1, *floR*, and the two sulfonamide resistance genes *sul*1 and *sul*2.

As expected, MICs determined for the tested AMPs protamine and OH-CATH-30 revealed species and isolate specificity (Table 4). Protamine MICs ranged between 15.63 µg/mL (*S. aureus* ATCC29213), 31.25 µg/mL (*E. coli* ATCC25922, *S. aureus* IMT37556), and 62.5 µg/mL (*E. coli* IMT37453). These MICs are in accordance with previously described MICs of protamine, which ranged from 7.8–31.25 µg/mL for different *S. aureus* strains and were determined with 50 µg/mL for *E. coli* in other studies [62,63].

MICs of OH-CATH-30 span a wider range, with MICs of 15.63 µg/mL (*E. coli* ATCC25922), 31.25 µg/mL (*E. coli* IMT37453), 250 µg/mL (*S. aureus* IMT37556), and ≥1000 µg/mL (*S. aureus* ATCC29213) (Table 4). OH-CATH-30 displayed inhibitory activity against the two *E. coli* strains, comparable to previously published MICs of OH-CATH-30 for different *E. coli* strains which ranged between 2 µg/mL and 16 µg/mL [55]. In comparison, the antimicrobial activity against the MRSA mastitis isolate was, with an MIC of 250 µg/mL, considerably low. In addition, the peptide lacked detectable antimicrobial activity in the tested concentration range (7.8–1000 µg/mL) against *S. aureus* ATCC29213 (Table 4). The relatively large difference between the MICs for the two *S. aureus* isolates could be due to differences in membrane structure described for MRSA and methicillin-sensitive *S. aureus* (MSSA) [64] that might influence AMP efficacy [65]. The determined MIC values were rounded up for the sensor experiments for better handling.

### 3.2. White Light Interferometry Results of Biofilm Thickness Measurements

**Figure 4:**
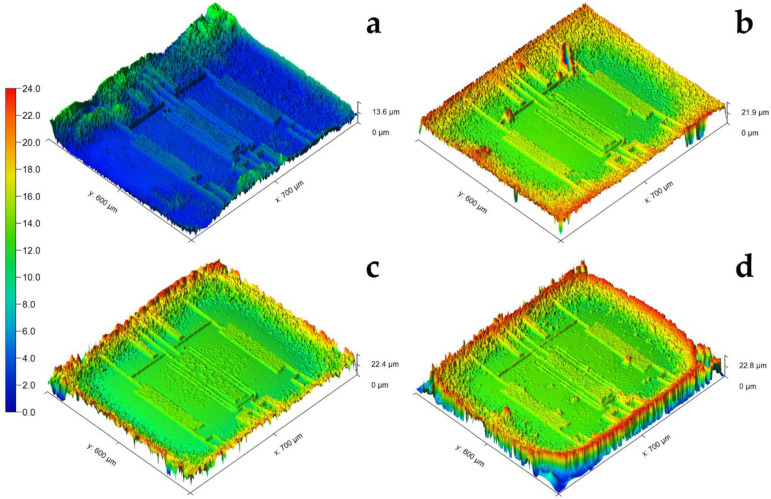
3D model of four representative white light interferometry measurements. Results of the reference strains *E. coli* ATCC25922 (**a**) and *S. aureus* ATCC29213 (**b**) as well as the mastitis isolates *E. coli* IMT37453 (**c**) and *S. aureus* IMT37556 (**d**) are shown. The dimensions of the sensor membrane are represented by the x-axis and y-axis. The z-axis represents the maximum height of the biofilm. The color scheme (dynamic) shows the different heights of adhering bacteria in µm.

White light interferometry was performed to verify the results obtained by the thermal sensor on bacterial growth in detail and to gain insights about the entire sensor surface. White light interferometry showed that the bacterial strains adhered and grew partially differently on the surface, as shown in Figure 4. Table 5 shows a comparable biofilm formation for the *S. aureus* reference strain and the mastitis isolates after 24 h. The exception was *E. coli* ATCC25922, which showed a thinner bacterial layer than the other strains.

Since the sensor membrane is flat, the bacterial adhesion on the entire surface should be almost identical. However, the sensor membrane structures, which were vapor deposited on the front side, were visible through the transparent membrane when using white light interferometry. Consequently, the light reflection by the structures led to measurement errors (phase jumps), interpreted by the software as an increased adherence of the bacteria, which shows up on the images as an apparent thickness variation, and can occur with thin membrane layers. The measurement errors were included in the calculation of the maximum and average thickness of adhering bacteria. The relation between the different isolates was not changed because the errors were identical for all measurements and the comparability of their adherence properties was still possible.

Compared to the values obtained for *E. coli* IMT37453 (Figure 4c, the biofilm formed by *E. coli* ATCC25922 (Figure 4a) showed a reduced max. height (approx. 50%). The mean height of *E. coli* ATCC25922 was approx. 75% less in comparison to *E. coli* IMT37453 (Table 5). Since all experiments were carried out according to the same protocol, this observation might indicate that *E. coli* ATCC25922 adherence is less effective with respect to the experimental growth conditions (medium and substrate).

### 3.3. Thermal Sensor Results

**Figure 5:**
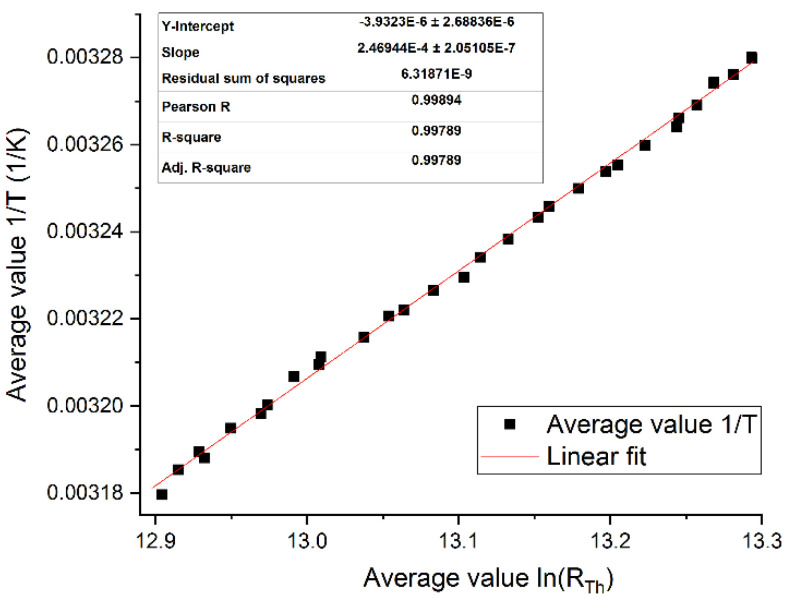
Calibration curve for amorphous Ge thermistor. Measured from 32–42 °C with a sampling rate of 1 Hz, the resistance is calculated based on the DC voltage and the temperature is measured with an NI USB-TC-01 (National Instruments, Austin, TX, USA) thermometer placed in the incubator. The Ge thermistor’s resistance and the temperature measured by the digital thermometer are recorded by a LabVIEW application. The incubator temperature has to be set manually by 0.1 °C every 30 s to minimize deviations. The average initial resistance at 32 °C is 593.9 kΩ. The slope of the logarithm of dimensionless thermistor resistance in relation to the inverse temperature is the second Steinhart–Hart coefficient (B), while the y-intercept is the first (A), both coefficients are dimensionless [66,67]. The calibration curve is calculated from the average values of three experiments. The temperature coefficient of resistance can be calculated through $\alpha =-1/{\left(B*T\right)}^{2}$. As the slope of the linear fit is 2.47 × 10^4^, the resulting α at 300 K is −4.49%/K. This is higher than the expected −2.2%/K, which is probably due to thermal stress between the membrane and the thermistor. For visual clarity, only every hundredth point is displayed.

The coefficient between the electrical and the thermal amplitude was determined by a series of calibration measurements (Figure 5). The relation between the logarithm of the resistance and the inverse temperature would ideally be linear, but the accuracy of the incubator and the slight temperature difference between the thermocouple and the sensor limited measurement accuracy.

Visible differences in precipitation and the corresponding rise in amplitude between DI water, MH1 medium, and MH1 medium with added AMPs were observed in the negative controls. DI water always showed the least increase, with the latter two being similar, depending on the applied dose of AMP. The signals in the negative control experiments with MH1 medium might be caused by the components of MH1 medium, especially casein hydrolysate (1.75%) and beef extract (30%) as well as the AMP itself, as shown by the dose dependency. The progression throughout the experiment showed a short rise followed by a constant amplitude during the measurement, indicating fast completion of precipitation compared to bacterial adherence.

The offset correction was conducted by setting the zero point at 60 min, as the amount of signal noise was too large before that time.

#### 3.3.1. Thermal Sensor Results of Inhibition of Protamine

**Figure 6:**
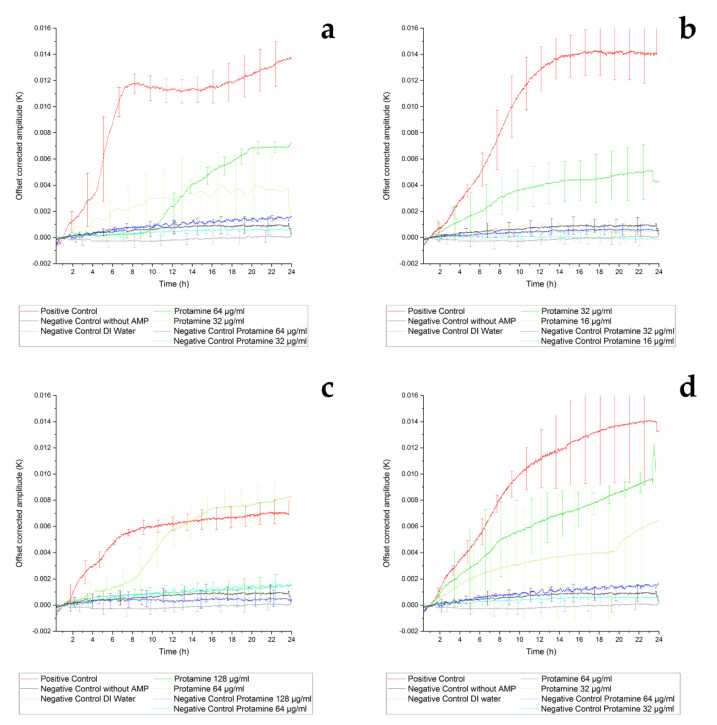
Representation of the thermal sensor measurement (at least n = 3). Different bacterial strains were incubated for 24 h at 37 °C in a thermal sensor setup. The amplitude was measured every second and filtered by a low-pass filter integrated in a lock-in amplifier. The offset was corrected by setting the zero point at 1 h. For clarity, the error bars were staggered. The displayed results were averaged from at least three experiments and the standard deviation is shown as well. The used strains were *E. coli* ATCC25922 (**a**) and IMT37453 (**c**) as well as *S. aureus* ATCC29213 (**b**) and IMT37556 (**d**). The strains were incubated in Mueller–Hinton-1 (MH1) medium, either pure medium for positive control (red), with a high or a low dose of the AMP protamine (both green). The negative controls (purple and blue) were also conducted with either dose to exclude the effect of AMP precipitation, as well as pure MH1 medium (black) and deionized (DI) water (gray) to exclude the effect of precipitation of medium.

Considering the thermal sensor measurement results, *E. coli* ATCC25922 showed strong growth without AMPs, in contrast to the results obtained using white light interferometry. Inhomogeneous bacterial growth on the surface might be an explanation for this discrepancy considering the sensor geometry, as the sensor is limited to measuring the adhesion between the heater and the thermistor. The large deviations in the amplitude measurement results were caused by inhomogeneous growth on the sensor surface, as well. In the case of white light interferometry, adhesion on the complete sensor membrane can be detected optically (Figure 4). Overall, the *E. coli* strain IMT37453 showed much weaker biofilm growth with the maximum amplitude of 7 mK being approximately 50% that of the other strains. The measurement results of *E. coli* IMT37453 were confirmed with more than 10 measurements with different thermal sensors, unlike the other thermal measurements. The large deviations at the beginning of the positive controls in Figure 6a,b were caused by unavoidable variations in the onset of biofilm growth and the limitation of the thermal sensor measurement.

The difference in bacterial growth between the *E. coli* strains might be due a different composition and structure of adhering bacteria. *E. coli* IMT37453 might form more water channels within the biofilm, which could be a possible explanation for the lower amplitude compared to *E. coli* ATCC25922 in the positive control as water has a higher thermal conductivity and volumetric heat capacity, resulting in a lower measurement signal [68].

Colonization of the sensor surface and biofilm growth of *E. coli* IMT37453 was almost completely inhibited by a concentration of 128 µg/mL protamine, while it was only partially and temporarily inhibited at 64 µg/mL of protamine, with strong growth starting at 8 h, overtaking even the positive control at 14 h and continuing to rise until 24 h. This indicates a promotion of biofilm growth, probably as a defensive mechanism or a consequence of the depletion of AMPs (Figure 6c) [13,69].

The growth of ATCC25922 was incompletely but permanently inhibited using a concentration of 32 µg/mL protamine, whereas the inhibition using 64 µg/mL protamine was initially stronger but lasted for 10 h only (Figure 6a). These results suggest that both protamine concentrations are insufficient to prevent adherence of all bacteria to the sensor surface. Attached bacteria may be not inhibited by the AMP and form a protective environment [13].

*E. coli* produces proteases directed against protamine, such as outer membrane proteases OmpT [70] and OmpP [71]. *E. coli* ATCC25922 and IMT37453 yield the *ompT* gene. The transcription of these enzymes might be induced only at high AMP concentrations. This phenomenon seems to be isolate specific. That might be an explanation for the higher inhibition of the bacterial growth when incubated with the lower AMP concentrations, which may not induce protease production (Figure 6a,c). The differences in bacterial growth between the *E. coli* strains might be due to a different composition and structure of adhering bacteria. Furthermore, cationic peptides like protamine can induce phenotypic changes in *S. aureus*, which might lead to a higher tolerance towards this AMP [72]. These phenotypic switches might be induced only at high AMP concentrations.

*S. aureus* reference strain ATCC29213 (Figure 6b) without AMPs also showed an attachment to the surface and adherence characteristics comparable with those recorded for *E. coli* ATCC25922 with a maximal amplitude of 14 mK, as seen in Figure 6a. Both reference strains (ATCC25922 and ATCC29213) and the isolate *S. aureus* IMT37556 showed partial inhibition of adherence using higher dose protamine and permanent inhibition with lower dose protamine. Attached bacteria may not be affected by the AMP and form a protective environment [13].

The discrepancy in the dose–activity relationship might indicate that adhering bacteria shield the cells residing in inner layers, and/or that bacteria recognize the presence of AMPs and increase their adherence as a response [69]. The *S. aureus* strains IMT37556 and ATCC29213 showed comparable growth for the positive controls. For both isolates, the higher concentrations of protamine tested (64 µg/mL and 32 µg/mL, respectively) inhibited the biofilm growth partially for the entire duration of the experiment, but the inhibition was a bit stronger at the lower dose again, which might be an indicator for a defense mechanism like the production of proteases (Figure 6d) [13]. Correa et al. used the peptides Pep19-2.5, Pep19-2.5LF, Pep19-4, and Pep19-4LF for their studies and recognized that at concentrations from 8 to 128 µg/mL, a lower dose led to a better antibacterial effect [73], which corresponds with the results gained in this study for protamine. To consolidate the results and the thesis of the defense mechanism, further experiments with additional protamine concentrations are needed.

The discrepancies between the results of the MIC determination and the thermal sensor measurements might be explained by the different detection limits of the two methods. This limit seems to be lower for the thermal sensor because of the higher sensitivity in contrast to MIC determination, so that even small amounts of bacteria adhering to the surface can be detected. The behavior of the different combinations of AMPs, AMP concentrations, and strains or isolates can lead to different results and must be regarded as an isolate-specific variable.

In addition, special inert microtiter plates made of polypropylene were used in the MIC determination protocol. Since the sensor is not made of such a material, it cannot be excluded that some of the AMPs interact with the surfaces [74] and are thus no longer available to destroy the adhering bacteria. Nevertheless, strain-specific higher inhibition of lower AMP concentrations was detected in the presented study. For this reason, experiments with additional higher and lower AMP concentrations may be necessary in the measurement setup of the sensor to guarantee complete inhibition of the bacterial adhesion and growth.

#### 3.3.2. Thermal Sensor Results of Inhibition of OH-CATH-30

**Figure 7:**
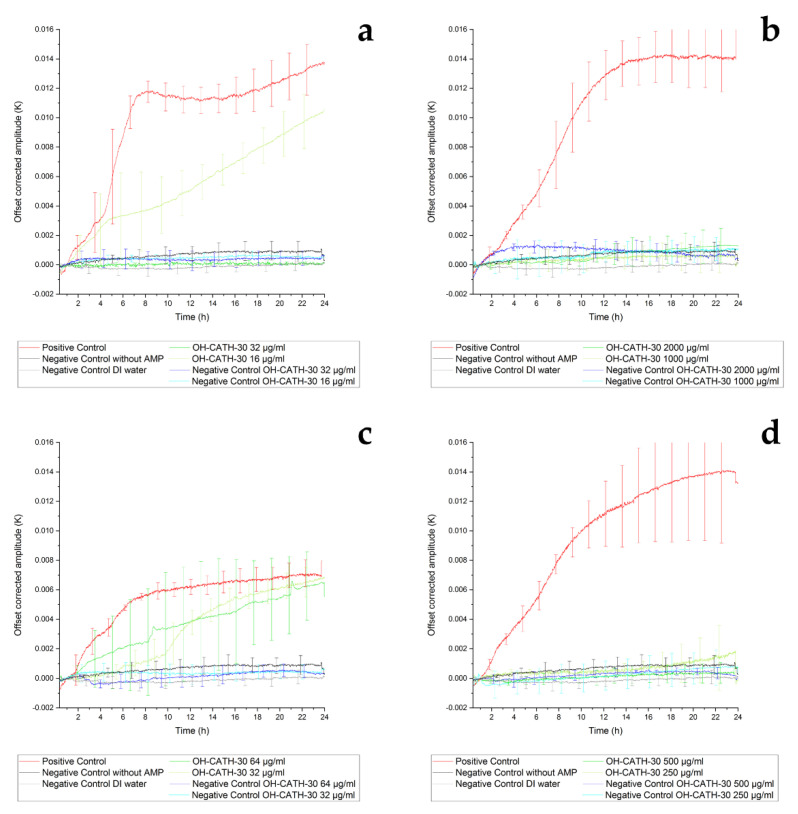
Representation of the thermal sensor measurement (at least n = 3). Different bacterial strains were incubated for 24 h at 37 °C in a thermal sensor setup. The amplitude was measured every second and filtered by a low-pass filter integrated in a lock-in amplifier. The offset was corrected by setting the zero point at 1 h. For clarity, the error bars are staggered. The displayed results were averaged from at least three experiments and the standard deviation is shown as well. The used strains were *E. coli* ATCC25922 (**a**) and IMT37453 (**c**) as well as *S. aureus* ATCC29213 (**b**) and IMT37556 (**d**). The strains were incubated in MH1 medium, either pure medium for positive control (red), with a high or a low dose of the AMP OH-CATH-30 (both green). The negative controls (purple and blue) were also conducted with either dose to exclude the effect of AMP precipitation, as well as pure MH1 medium (black) and DI water (gray) to exclude the effect of precipitation of medium.

The *E. coli* ATCC25922 experiments with 32 µg/mL of OH-CATH-30 showed permanent inhibition of the bacterial adhesion and biofilm formation while the growth at 16 µg/mL was partially inhibited (Figure 7a). The large deviations at the beginning of the positive controls in Figure 7a,b were caused by unavoidable variations in the onset of biofilm growth and the limitation of the thermal sensor measurement. Both OH-CATH-30 concentrations tested (64 µg/mL and 32 µg/mL) were not sufficient to inhibit the adherence of *E. coli* IMT37453 in this particular setup (Figure 7c).

The growth and surface colonization of *S. aureus* IMT37556 (250 µg/mL and 500 µg/mL) and ATCC29213 (1000 µg/mL and 2000 µg/mL) were completely inhibited at both concentrations of OH-CATH-30 tested, as seen in Figure 7b,d. The effect of this AMP might be more efficient at these high concentrations against members of Gram-positive species, a hypothesis that needs to be verified in further studies. The differences between the cell walls of Gram-positive and -negative bacteria according the murein layer and the composition might be an explanation for the higher MIC values of the two *S. aureus* strains compared the tested *E. coli* [75]. Furthermore, Gram-positive bacteria are able to partially neutralize their cell wall to resist AMPs [76,77]. The variations between the MIC determination and the sensor measurement are due to the different detection limits.

In this setting, OH-CATH-30 could be identified as the more efficient of the two analyzed AMPs, especially regarding its efficacy at high concentrations against *S. aureus* strains. Protamine does not interact with the bacterial membrane, resulting in its disruption, as OH-CATH-30 does, but penetrates the bacterial cell and possesses an intracellular mode of action [78]. These two completely different modes of action seem to lead to different efficiencies against the tested strains. The structure of the cell wall of both *S. aureus* strain isolates seems to lead to an increased tolerance against OH-CATH-30, which might be an explanation for the high MIC values of these two strains.

Certain fragments of an AMP can be sufficient to lyse bacterial membranes. This can enhance the bactericidal effect and reduce the molecular weight and therefore the cost of production due to the shortened primary sequence.

In the case of the AMP OH-CATH-30, the first four N-terminal amino acids of the original peptide were removed to reduce the hemolytic effect without affecting its activity against bacteria. However, the removal of the C-terminal amino acids leads to a loss of bactericidal activity. The AMP fragment OH-CATH-30 (amino acid 5–34) showed the highest bactericidal activity against 11 tested bacterial strains, especially ATCC25922 [55]. Further experiments with modified and optimized AMPs or combinations of different AMPs could lead to a higher antimicrobial activity against a broad panel of different bacterial species.

Another promising approach would be to use the AMP Magainin H2 because of its higher degree of hydrophobicity than Magainin 2. It also showed a strong permeabilization activity on lipid bilayers. First, the lateral expansion of the membrane induced by the interaction with the AMP is inhibited. Further, it proceeds to create new bilayer regions with many defects in the membrane [79,80].

## 4. Conclusions

A new and promising thermal sensor connection setup that is not affected by ionic strength can be used for measurements of bacterial adhesion and biofilm formation in real time [44].

The thermal sensor measurement was performed for 24 h at 37 °C under static conditions in the sample chamber of the thermal sensor connection setup. White light interferometry was used for optical control to determine the amount of bacterial adhesion and demonstrated the formation of a biofilm after 24 h on the sensor surface. The thermal measurement allows the detection of structural differences in a bacterial population and adherence on the sensor membrane. Structural differences in the biofilm architecture can be identified with this system in real time. In the case of the thermal measurements, the *E. coli* strain IMT37453 showed much weaker biofilm growth than the other strains. The different bacterial growth between the *E. coli* strains might be due a different composition and structure of adhering bacteria. Further, it was shown the lower concentrations of the AMP protamine had a stronger effect on bacterial adhesion of three of the tested isolates. The effect could not be detected for the AMP OH-CATH-30. High concentrations of protamine might enhance the secretion of specific proteases. These very interesting results will be part of future studies. Furthermore, the tested concentrations of OH-CATH-30 were sufficient to inhibit the growth and adhesion of *S. aureus*.

Overall, the system can be used not only for the investigation of the effect of various biological or chemical disinfectants but also for the characterization of the adhesion of different bacterial species/strains or mixed cultures and different media.

The authors have searched for comparable technologies that can detect the effect of AMPs on bacteria in real time, but could not find any equivalent experiments with AMPs in solution utilizing other sensor technologies.

## 5. Outlook

In one of the next development stages, it would be useful to integrate the sensor into the wall or lid of the sample chamber to distinguish between adhering bacteria and other influences such as sedimentation of non-motile bacteria or components of the medium in the current setup, as any influence of sedimentation would be mostly eliminated if the sensor was operated in a vertical orientation.

Furthermore, the sensor could be integrated into the wall of a pipe in milking systems or subsystems to measure the growth of adhering bacteria and subsequent biofilm formation under flow conditions and for real-time monitoring of potential pathogen adhesion. Dairy industry equipment is extremely sensitive to all kinds of microorganisms and remaining milk residue in the pipeline provides a source of nutrients and favors the survival of the biofilm. This is the main reason for the contamination of milk storage tanks and milk process lines [81].

Additionally, the sensor can also monitor contaminants such as limestone scale or other deposits, which provides significant advantages for its use in the food and dairy industry.

Another option would be to manufacture the entire evaluation electronics system as an integrated circuit on a wafer with the sensor, allowing, for example, the installation of a separate sensor in each well of a 96-well microwell plate and thus parallelization of the experiments.

A thin polymer coating also has the advantage that the top side could be used for the measurements and would no longer have to be measured in the cavity, which is currently not possible due to pinholes in the PECVD silicon nitride. A measurement on the upper side would have the benefit that the sensitivity of the sensor is less restricted by the membrane since the thermal waves no longer have to penetrate it on their way into the medium.

It is also possible to manufacture the chip from a polymer such as polyetherimide, except for heaters, conductors, and thermistors. These changes would be useful due to increased toughness and lower thermal conductivity of the membrane.

Another option is to integrate additional sensors such as impedimetric or optical sensors on the chip. The thermal sensor measures different parameters than an impedimetric sensor, which is why being able to detect both types of parameters may result in synergy. This allows a better understanding of the composition and structure of the biofilm.

Finally, it should also be possible to measure the biofilm formation on a physical plasma-generated antibacterial surface coated with AMPs embedded in a nanocomposite.

## Figures and Tables

**Table 1:** State of the art bacterial adherence monitoring. Summary with advantages and disadvantages of the measurement methods.

Measurement Method	+ Advantages	− Disadvantages	Ref.
Surface acoustic waves (SAWs)	+ high sensitivity + easy to produce + low cost	− limited height − measurement − limited reusability	[30,31,32]
Electrochemical impedance spectroscopy (EIS)	+ non-invasive + easy to integrate	− no long-term stability − limited reusability − baseline drift − low sensitivity	[34,35,36]
Quartz crystal microbalance (QCM)	+ high sensitivity + label free	− temperature sensitive − affected by medium turbidity	[35,37]
Isothermal micro-calorimetry (IMC)	+ metabolic information + non-invasive + non-destructive	− long settling time − heat source indistinguishable	[36,38]
Time-invariant heat transfer	+ easy to prepare	− requires knowledge about thermal properties	[39]
Optical detection	+ easy to handle	− no thickness measurement − time consuming − affected by medium turbidity	[35,40,41,42]

**Table 2:** Tested antibiotics and concentration range.

Antibiotic	Concentration in µg/mL
Penicillin G	0.125–8
Ampicillin	4–16
Cefazolin	4–32
Cefoperazone	2–16
Cefquinome	1–8
Oxacillin	1–4
Pirlimycin	1–4
Erythromycin	0.125–4
Marbofloxacin	0.25–2
Amoxicillin/clavulanic acid	4/2–32/16
Kanamycin/cefalexin	4/0.4–32/3.2

**Table 3:** Sequence and molecular properties of used antimicrobial peptides (AMPs).

AMP	Sequence (Primary Structure) Molecular Weight (g/mol) Degree of Purity (%)
Protamine	MPRRRRSSSRPVRRRRRPRVSRRRRRRGGRRRR
	4381.24
	96.94
OH-CATH-30	KFFKKLKNSVKKRAKKFFKKPRVIGVSIPF
	3595.55
	99.44

**Table 4:** Results of AMP minimum inhibitory concentration (MIC) experiments. Results determined in at least three independent repetitions.

Bacterial Strains	Protamine MIC in µg/mL	OH-CATH-30 MIC in µg/mL
*E. coli (ATCC25922)*	31.25	15.63
*S. aureus (ATCC29213)*	15.63	≥1000
*E. coli (IMT37453)*	62.5	31.25
*S. aureus (IMT37556)*	31.25	250

**Table 5:** Representation of the z-axis detected after 24 h of incubation by white light interferometry measurement. Mean values, their standard deviations, and the maximum level of biofilm formation of *n* = 4 measurements (z-axis). The values are given in µm.

Bacterial Strains	Max. Value (Z-axis) in µm	Mean Value (Z-axis) in µm
*E. coli (ATCC25922)*	12.14 ± 1.80	2.66 ± 1.12
*S. aureus (ATCC29213)*	16.58 ± 7.12	8.61 ± 5.07
*E. coli (IMT37453)*	21.48 ± 2.06	11.70 ± 1.43
*S. aureus (IMT37556)*	22.31 ± 1.00	12.97 ± 0.58

## Data Availability

The data presented in this study are available on request from the corresponding author.

## References

[1] Gaynes R. (2017). The Discovery of Penicillin—New Insights After More Than 75 Years of Clinical Use. Emerg. Infect. Dis..

[2] Bowler P.G. (2018). Antibiotic resistance and biofilm tolerance: A combined threat in the treatment of chronic infections. J. Wound Care.

[3] Cassini A., Högberg L.D., Plachouras D., Quattrocchi A., Hoxha A., Simonsen G.S., Colomb-Cotinat M., E Kretzschmar M., Devleesschauwer B., Cecchini M. (2019). Attributable deaths and disability-adjusted life-years caused by infections with antibiotic-resistant bacteria in the EU and the European Economic Area in 2015: A population-level modelling analysis. Lancet Infect. Dis..

[4] Marshall B.M., Levy S.B. (2011). Food Animals and Antimicrobials: Impacts on Human Health. Clin. Microbiol. Rev..

[5] Antão E.-M., Vincze S., Hanke R., Klimmek L., Suchecka K., Lübke-Becker A., Wieler L.H. (2018). Antibiotic resistance, the 3As and the road ahead. Gut Pathog..

[6] Hall-Stoodley L., Costerton J.W., Stoodley P. (2004). Bacterial biofilms: From the Natural environment to infectious diseases. Nat. Rev. Genet..

[7] da Silva Araújo P.A. (2014). Biofilm Control with Antimicrobial Agents: The Role of the Exopolymeric Matrix.

[8] Jamal M., Ahmad W., Andleeb S., Jalil F., Imran M., Nawaz M.A., Hussain T., Ali M., Rafiq M., Kamil M.A. (2018). Bacterial biofilm and associated infections. J. Chin. Med. Assoc..

[9] Mah T.-F.C., O’Toole A.G. (2001). Mechanisms of biofilm resistance to antimicrobial agents. Trends Microbiol..

[10] Stoodley P., Sauer K., Davies D.G., Costerton J.W. (2002). Biofilms as Complex Differentiated Communities. Annu. Rev. Microbiol..

[11] Lewis K. (2001). Riddle of Biofilm Resistance. Antimicrob. Agents Chemother..

[12] Adlhart C., Verran J., Azevedo N.F., Olmez H., Keinänen-Toivola M.M., Gouveia I., Melo L.F., Crijns F. (2018). Surface modifications for antimicrobial effects in the healthcare setting: A critical overview. J. Hosp. Infect..

[13] Stewart P.S., Costerton J.W. (2001). Antibiotic resistance of bacteria in biofilms. Lancet.

[14] Melchior M., Vaarkamp H., Fink-Gremmels J. (2006). Biofilms: A role in recurrent mastitis infections?. Veter. J..

[15] Mainau E., Temple D., Manteca X. (2014). Welfare issues related to mastitis in dairy cows. Farm Anim. Welf. Educ. Cent..

[16] Madsen J.S., Burmølle M., Hansen L.H., Sørensen S.J. (2012). The interconnection between biofilm formation and horizontal gene transfer. FEMS Immunol. Med. Microbiol..

[17] Matsuzaki K. (2001). Why and how are peptide-lipid interactions utilized for self defence?. Biochem. Soc. Trans..

[18] Reddy K., Yedery R., Aranha C. (2004). Antimicrobial peptides: Premises and promises. Int. J. Antimicrob. Agents.

[19] Rapsch K., Bier F.F., Tadros M., Von Nickisch-Rosenegk M. (2014). Identification of Antimicrobial Peptides and Immobilization Strategy Suitable for a Covalent Surface Coating with Biocompatible Properties. Bioconjugate Chem..

[20] Cole A.M., Ganz T. (2000). Human Antimicrobial Peptides: Analysis and Application. Biotechniques.

[21] Hancock R.E., Diamond G. (2000). The role of cationic antimicrobial peptides in innate host defences. Trends Microbiol..

[22] Chung P.Y., Khanum R. (2017). Antimicrobial peptides as potential anti-biofilm agents against multidrug-resistant bacteria. J. Microbiol. Immunol. Infect..

[23] Ebenhan T., Gheysens O., Kruger H.G., Zeevaart J.R., Sathekge M.M. (2014). Antimicrobial Peptides: Their Role as Infection-Selective Tracers for Molecular Imaging. BioMed. Res. Int..

[24] Park S.-C., Park Y., Hahm K.-S. (2011). The Role of Antimicrobial Peptides in Preventing Multidrug-Resistant Bacterial Infections and Biofilm Formation. Int. J. Mol. Sci..

[25] Nicolas P. (2009). Multifunctional host defense peptides: Intracellular-targeting antimicrobial peptides. FEBS J..

[26] Zasloff M. (2002). Antimicrobial peptides of multicellular organisms. Nat. Cell Biol..

[27] Mahlapuu M., Håkansson J., Ringstad L., Björn C. (2016). Antimicrobial Peptides: An Emerging Category of Therapeutic Agents. Front. Cell. Infect. Microbiol..

[28] O’Toole G.A. (2011). Microtiter Dish Biofilm Formation Assay. J. Vis. Exp..

[29] Neu T.R., Lawrence J.R. (2015). Innovative techniques, sensors, and approaches for imaging biofilms at different scales. Trends Microbiol..

[30] Kim Y.W., Meyer M.T., Berkovich A., Subramanian S., Iliadis A.A., Bentley W.E., Ghodssi R. (2016). A surface acoustic wave biofilm sensor integrated with a treatment method based on the bioelectric effect. Sens. Actuators A Phys..

[31] Kim Y.W., Sardari S.E., Meyer M.T., Iliadis A.A., Wu H.C., Bentley W.E., Ghodssi R. (2012). An ALD aluminum oxide passivated Surface Acoustic Wave sensor for early biofilm detection. Sens. Actuators B Chem..

[32] Schmid L., Franke T. (2018). Real-time size modulation and synchronization of a microfluidic dropmaker with pulsed surface acoustic waves (SAW). Sci. Rep..

[33] Pires L., Sachsenheimer K., Kleintschek T., Waldbaur A., Schwartz T., Rapp B.E. (2013). Online monitoring of biofilm growth and activity using a combined multi-channel impedimetric and amperometric sensor. Biosens. Bioelectron..

[34] Ghafar-Zadeh E., Sawan M., Shabani A., Zourob M., Chodavarapu V. Bacteria growth monitoring through an on-chip capacitive sensor. Proceedings of the 2008 IEEE 14th International Mixed-Signals Sensors, and Systems Test Workshop.

[35] Reipa V., Almeida J., Cole K.D. (2006). Long-term monitoring of biofilm growth and disinfection using a quartz crystal microbalance and reflectance measurements. J. Microbiol. Methods.

[36] Lerchner J., Wolf A., Buchholz F., Mertens F., Neu T., Harms H., Maskow T. (2008). Miniaturized calorimetry—A new method for real-time biofilm activity analysis. J. Microbiol. Methods.

[37] Zhang Q., Cui H., Xiong X., Chen J., Wang Y., Shen J., Luo Y., Chen L. (2017). QCM-nanomagnetic beads biosensor for lead ion detection. Analyst.

[38] Mariana F., Buchholz F., Lerchner J., Neu T.R., Harms H., Maskow T. (2013). Chip-calorimetric monitoring of biofilm eradication with antibiotics provides mechanistic information. Int. J. Med. Microbiol..

[39] Stenberg M., Stemme G., Kittilsland G., Pedersen K. (1988). A silicon sensor for measurement of liquid flow and thickness of fouling biofilms. Sens. Actuators.

[40] Fischer M., Wahl M., Friedrichs G. (2012). Design and field application of a UV-LED based optical fiber biofilm sensor. Biosens. Bioelectron..

[41] Kwak Y.H., Lee J., Lee J., Kwak S.H., Oh S., Paek S.-H., Ha U.-H., Seo S. (2014). A simple and low-cost biofilm quantification method using LED and CMOS image sensor. J. Microbiol. Methods.

[42] Kristich C.J., Li Y.-H., Cvitkovitch D.G., Dunny G.M. (2004). Esp-Independent Biofilm Formation by *Enterococcus faecalis*. J. Bacteriol..

[43] Zibaii M.I., Kazemi A., Latifi H., Azar M.K., Hosseini S.M., Ghezelaiagh M.H. (2010). Measuring bacterial growth by refractive index tapered fiber optic biosensor. J. Photochem. Photobiol. B Biol..

[44] Reyes-Romero D., Behrmann O., Dame G., Urban G. (2014). Dynamic thermal sensor for biofilm monitoring. Sens. Actuators A Phys..

[45] Ernst H., Jachimowicz A., Urban G. (2001). Dynamic thermal sensor—Principles in MEMS for fluid characterization. IEEE Sens. J..

[46] Ernst H., Jachimowicz A., Urban G.A. (2002). High resolution flow characterization in Bio-MEMS. Sens. Actuators A Phys..

[47] Çubukçu A.S., Romero D.F.R., Urban G.A. (2014). A dynamic thermal flow sensor for simultaneous measurement of thermal conductivity and flow velocity of gases. Sens. Actuators A Phys..

[48] Kuntner J., Kohl F., Jakoby B. (2006). Simultaneous thermal conductivity and diffusivity sensing in liquids using a micromachined device. Sens. Actuators A Phys..

[49] Beigelbeck R., Nachtnebel H., Kohl F., Jakoby B. (2011). A novel measurement method for the thermal properties of liquids by utilizing a bridge-based micromachined sensor. Meas. Sci. Technol..

[50] Romero D.R., Kogan K., Cubukcu A.S., Urban G.A. (2013). Simultaneous flow and thermal conductivity measurement of gases utilizing a calorimetric flow sensor. Sens. Actuators A Phys..

[51] Watts J.L. (2013). Performance Standards for Antimicrobial Disk and Dilution Susceptibility Tests for Bacteria Isolated from Animals: Approved Standard.

[52] Feßler A., Böttner A., Fehr M., Kasper H., Kehrenberg C., Kietzmann M. (2017). Mikrotiterplattenlayouts für Kleintiere, Großtiere und Mastitis: Aktualisierung der Layouts des DVG-Arbeitskreises Antibiotikaresistenz. Dtsch. Tierärzteblatt.

[53] Miller B.F., Abrams R., Dorfman A., Klein M. (1942). Antibacterial Properties of Protamine and Histone. Am. Assoc. Adv. Sci. Stable.

[54] Tadros M. (2009). Method for the Production of Protamine. U.S. Patent.

[55] Zhang Y., Zhao H., Yu G.-Y., Liu X.-D., Shen J.-H., Lee W.-H., Zhang Y. (2010). Structure–function relationship of king cobra cathelicidin. Peptides.

[56] Wiegand I., Hilpert K., Hancock R.E.W. (2008). Agar and broth dilution methods to determine the minimal inhibitory concentration (MIC) of antimicrobial substances. Nat. Protoc..

[57] Bankevich A., Nurk S., Antipov D., Gurevich A.A., Dvorkin M., Kulikov A.S., Lesin V.M., Nikolenko S.I., Pham S., Prjibelski A.D. (2012). SPAdes: A New Genome Assembly Algorithm and Its Applications to Single-Cell Sequencing. J. Comput. Biol..

[58] Seemann T. (2014). Prokka: Rapid Prokaryotic Genome Annotation. Bioinformatics.

[59] Munby M., Fujiki J., Aoki K., Kawaguchi C., Nakamura K., Nakamura T., Sasaki M., Sato T., Usui M., Sawa H. (2020). Whole-Genome Sequence of Fluoroquinolone-Resistant Escherichia coli HUE1, Isolated in Hokkaido, Japan. Microbiol. Resour. Announc..

[60] Manson J.-A.E., Seferis J.C. (1989). Autoclave Processing of PEEK/Carbon Fiber Composites. J. Thermoplast. Compos. Mater..

[61] Udachan L.A., Shivaprasad S.M., Ashrit P.V., Angadi M.A. (1980). Electrical resistivity and temperature coefficient of resistance of vacuum evaporated thin chromium films. Phys. Status Solidi.

[62] Gottlieb C.T., Thomsen L.E., Ingmer H., Mygind P.H., Kristensen H.-H., Gram L. (2008). Antimicrobial peptides effectively kill a broad spectrum of *Listeria monocytogenes* and *Staphylococcus aureus* strains independently of origin, sub-type, or virulence factor expression. BMC Microbiol..

[63] Hansen L.T., Gill T. (2000). Solubility and antimicrobial efficacy of protamine on *Listeria monocytogenes* and *Escherichia coli* as influenced by pH. J. Appl. Microbiol..

[64] Pinho M.G., De Lencastre H., Tomasz A. (2001). An acquired and a native penicillin-binding protein cooperate in building the cell wall of drug-resistant staphylococci. Proc. Natl. Acad. Sci. USA.

[65] Peschel A., Jack R.W., Otto M., Collins L.V., Staubitz P., Nicholson G., Kalbacher H., Nieuwenhuizen W.F., Jung G., Tarkowski A. (2001). *Staphylococcus aureus* Resistance to Human Defensins and Evasion of Neutrophil Killing via the Novel Virulence Factor Mprf Is Based on Modification of Membrane Lipids with l-Lysine. J. Exp. Med..

[66] Reyes Romero D.F. (2014). Development of a Medium Independent Flow Measurement Technique Based on Oscillatory Thermal Excitation.

[67] Steinhart J.S., Hart S.R. (1968). Calibration curves for thermistors. Deep Sea Res. Oceanogr. Abstr..

[68] Characklis W.G., Nevimons M.J., Picologlou B.F. (1981). Influence of Fouling Biofilms on Heat Transfer. Heat Transf. Eng..

[69] Li M., Cha D.J., Lai Y., Villaruz A.E., Sturdevant D.E., Otto M. (2007). The antimicrobial peptide-sensing system aps of *Staphylococcus aureus*. Mol. Microbiol..

[70] Stumpe S., Schmid R., Stephens D.L., Georgiou G., Bakker E.P. (1998). Identification of OmpT as the Protease That Hydrolyzes the Antimicrobial Peptide Protamine before It Enters Growing Cells of *Escherichia coli*. J. Bacteriol..

[71] Hwang B.-Y., Varadarajan N., Li H., Rodriguez S., Iverson B.L., Georgiou G. (2006). Substrate Specificity of the *Escherichia coli* Outer Membrane Protease OmpP. J. Bacteriol..

[72] Koo S.-P., Bayer A.S., Yeaman M.R. (2001). Diversity in Antistaphylococcal Mechanisms among Membrane-Targeting Antimicrobial Peptides. Infect. Immun..

[73] Correa W., Heinbockel L., Behrends J., Kaconis Y., Barcena-Varela S., Gutsmann T., Mauss K., Schürholz T., Schromm A.B., De Tejada G.M. (2019). Antibacterial action of synthetic antilipopolysaccharide peptides (SALP) involves neutralization of both membrane-bound and free toxins. FEBS J..

[74] Li Y., Wei S., Wu J., Jasensky J., Xi C., Li H., Xu Y., Wang Q., Marsh E.N.G., Brooks C.L. (2015). Effects of Peptide Immobilization Sites on the Structure and Activity of Surface-Tethered Antimicrobial Peptides. J. Phys. Chem. C.

[75] Assoni L., Milani B., Carvalho M.R., Nepomuceno L.N., Waz N.T., Guerra M.E.S., Converso T.R., Darrieux M. (2020). Resistance Mechanisms to Antimicrobial Peptides in Gram-Positive Bacteria. Front. Microbiol..

[76] Maria-Neto S., De Almeida K.C., Macedo M.L.R., Franco O.L. (2015). Understanding bacterial resistance to antimicrobial peptides: From the surface to deep inside. Biochim. Biophys. Acta (BBA) Biomembr..

[77] Henderson J.C., Fage C.D., Cannon J.R., Brodbelt J.S., Keatinge-Clay A.T., Trent M.S. (2014). Antimicrobial Peptide Resistance of Vibrio cholerae Results from an LPS Modification Pathway Related to Nonribosomal Peptide Synthetases. ACS Chem. Biol..

[78] Aspedon A., Groisman E.A. (1996). The antibacterial action of protamine: Evidence for disruption of cytoplasmic membrane energization in *Salmonella typhimurium*. Microbiology.

[79] Marín-Medina N., Mescola A., Alessandrini A. (2018). Effects of the peptide Magainin H2 on Supported Lipid Bilayers studied by different biophysical techniques. Biochim. Biophys. Acta (BBA) Biomembr..

[80] Mescola A., Marín-Medina N., Ragazzini G., Accolla M., Alessandrini A. (2019). Magainin-H2 effects on the permeabilization and mechanical properties of giant unilamellar vesicles. J. Colloid Interface Sci..

[81] Vishwakarma V. (2020). Impact of environmental biofilms: Industrial components and its remediation. J. Basic Microbiol..

